# Non-invasive epidermis sampling for DNA methylation-based prediction of skin cancer phenotypes

**DOI:** 10.1038/s41698-026-01302-7

**Published:** 2026-01-27

**Authors:** Manuel Rodríguez-Paredes, Yan Feng, Oliver Gilliam, Katrin Wegner, Günter Raddatz, Elke Grönniger, Frank Lyko

**Affiliations:** 1 Division of Epigenetics, DKFZ-ZMBH Alliance, German Cancer Research Center, Heidelberg, Germany; 2 Research & Development, Beiersdorf AG, Hamburg, Germany

**Keywords:** Biomarkers, Cancer, Computational biology and bioinformatics, Genetics, Molecular biology

## Abstract

The epidermis is uniquely exposed to the effects of environmental factors, such as ultraviolet radiation, which induce progressive genetic and epigenetic modifications contributing to aging and the onset of keratinocyte carcinomas. DNA methylation is the best-characterized epigenetic modification and a valuable biomarker for assessing epidermal health. However, broad screening approaches have been hindered by the limited quantity and quality of the genomic DNA obtained from the epidermis and the resulting need for invasive sampling methods. Here we describe an integrated method that enables the non-invasive sampling of epidermal DNA for subsequent analysis by DNA methylation microarrays. This procedure combines a gel-based adhesive tape for keratinocyte collection, a robust gDNA extraction protocol and a curated selection of microarray probes optimized for low-input DNA conditions. Analysis of >100 corresponding methylomes demonstrates that our approach can be used for both the training of epigenetic clocks capable of predicting epidermal age with high accuracy, as well as the investigation of various DNA methylation-based biomarkers relevant to keratinocyte cancer development. These findings underscore the potential of our method for broad and non-invasive skin health assessment and cancer prevention strategies.

## Introduction

The skin, which constitutes the largest organ of the human body, fulfills multiple roles essential for human health and survival^1^. Primarily, it serves as a protective barrier against environmental (e.g., temperature variations, ultraviolet radiation (UVR)), chemical (e.g., allergens, irritants), and microbial (bacterial, viral, fungal) threats^2,3^. Additionally, the skin prevents water loss, thereby averting desiccation^2,3^. Structurally, the skin comprises two main layers: the epidermis and the dermis, both of which are supported by an underlying adipose tissue layer sometimes referred to as the hypodermis^1^. Organized as a stratified squamous epithelium, the epidermis contains epidermal stem cells in its *stratum basale* that undergo differentiation while migrating through other *strata*, including *spinosum*, *granulosum*, *lucidum* and *corneum*, to replace the dead, anucleated and keratinized (squamous) keratinocytes that are shed from the surface of the skin^4–6^. In this regard, it is important to note that epidermal keratinocytes begin to die already in the *stratum granulosum*.

As the outermost layer, the epidermis is most exposed to the environment, with UVR exerting the most significant impact. UVR-induced cell damage, both genetic and epigenetic, is key to skin aging and constitutes the greatest risk factor for the development of keratinocyte cancers (KCs)^7–9^. KCs, including basal cell carcinoma (BCC) and cutaneous squamous cell carcinoma (cSCC), are the most prevalent tumors among fair-skinned populations, with millions of cases reported annually in Europe and the USA^10,11^. Furthermore, epidemiological estimates have suggested that 69% of the Australian population will develop at least one KC during their lifetime^12^. The high prevalence of these cancers, coupled with substantial healthcare costs and a rising incidence through increased life expectancy and unprotected sun exposure, underscores their significance as a major public health concern^13^.

DNA methylation is an epigenetic mechanism in which a methyl group is covalently added to cytosines at the fifth carbon of their pyrimidine ring, predominantly in a CG dinucleotide context^14^. The process is mediated by a set of DNA methyltransferases in response to external signals, both at the cellular (e.g., hormones, cell-cell interactions) and the organismal level (diet, lifestyle)^15^. It generates methylation patterns in the genome that, among other functions, influence gene expression by regulating chromatin accessibility in key regions, such as promoters, enhancers or gene bodies^14,15^. Consequently, DNA methylation is crucial not only for defining cell identity and ensuring proper cellular function, but also for cell differentiation and the maintenance of tissue homeostasis, which holds particular significance in the epidermis^8,16^. As for other tissues, epidermal DNA methylation profiles are known to undergo significant changes during aging and tumorigenesis. Known changes include CpG-island promoter hypermethylation events leading to the silencing of various genes, hypomethylation of the lamina-associated domains (LADs) that favor genomic instability, and a general erosion of methylation patterns, known as epigenetic drift^8^. Cancer-specific methylation changes occur early in the disease continuum, as they are already present in precursor lesions^17^.

DNA methylation patterns are also valuable biomarkers of the general health status of tissues. This is illustrated by the development of epigenetic clocks^18^, which are computational algorithms originally developed to estimate the chronological age of biological samples based on age-related methylation changes. We and others have previously pioneered the development of epigenetic clocks for human skin^19–21^. Additionally, second-generation clocks have been established that are capable of predicting factors closely related to cancer risk, such as the minimal erythemal dose and the rate of cellular mitosis^22–24^. However, the lack of suitable non-invasive methods for skin sampling has thus far precluded the broader use of methylation-based diagnostics for skin analysis.

Tape stripping represents a rapid and non-invasive procedure with significant potential for obtaining biomolecules from human skin^25–27^. This technique involves the use of a variable number of small adhesive strips to detach cells from the outermost layers of the tissue. Despite its lack of standardization, the method has proven to be adaptable for the study of the cutaneous microbiome, as well as for analyses targeting molecules such as lipids, RNA, and proteins^25–27^. Unlike gDNA, these biomolecules can be retrieved without significant degradation from cells in the epidermal *strata granulosum* and *corneum*. To date, only one study has successfully adapted tape stripping to analyze epidermal methylomes^28^. However, the protocol required the use of magnetic beads for DNA isolation, enzymatic treatment instead of the standard sodium bisulfite for DNA conversion, and PCR-based DNA amplification, which is known to introduce artifacts into DNA methylation analysis^29^. Finally, methylation patterns could only be analyzed by sequencing, which is relatively expensive and requires extensive computational resources for data analysis.

The availability of methylation microarrays has greatly facilitated DNA methylation analysis of human samples and can be considered the gold standard for clinical epigenetic diagnostics^30–32^. This prominence is supported by numerous repositories containing datasets from various conditions and diseases^33^, along with the development of tailored computational packages that facilitate both direct and indirect analyses of the methylation data^33–38^. In fact, most epigenetic clocks have been trained with datasets provided by these microarrays. Interestingly, it has been shown recently that ultra-low quantities of gDNA can be successfully analyzed without PCR amplification on *Infinium* MethylationEPIC arrays^30^, which raised the possibility that DNA from tape stripping could also be analyzed using this platform.

Here, we introduce a method that integrates a low number of advanced adhesive strips —based on a blend of a polyacrylate adhesive coating and a polyurethane film—with an optimized DNA extraction protocol and a computational workflow that enables the combination of tape stripping with DNA methylation microarrays. This method, which we termed TapeLift, allowed us to non-invasively generate high-quality epidermal methylomes from >100 donors. Data analysis showed a high degree of similarity between TapeLift methylomes and methylomes acquired by conventional, invasive methods. Furthermore, we verified that these epidermal methylomes are suitable for training DNA methylation clocks, predicting donor ages with a mean error of 4.3 years. Finally, we analyzed TapeLift datasets for various skin cancer markers to illustrate the potential of the method for the development of KC risk predictors. Our findings thus open a wide field of potential applications, ranging from the quantitative analysis of anti-aging interventions to skin cancer prevention.

## Results

### Generation of high-quality epidermal methylomes using the TapeLift protocol

Obtaining sufficient quantities of high-quality gDNA from the outermost layers of the epidermis presents a significant challenge, as keratinocytes lose their nuclei during desquamation. To enable the non-invasive collection of intact gDNA, we used an advanced polyacrylate adhesive coating on a polyurethane film to provide an optimal balance of yield and quality, particularly when applied to the forehead, which we selected as a representative area for sun-exposed skin. When an optimized gDNA isolation protocol (see Methods for details) was applied to four adhesive tapes that were consecutively taken from the same area of the forehead of 105 healthy donors of all ages and both genders, we obtained an average DNA yield of 49 ng (Table S1). This is significantly more than the minimum amount previously tested successfully with *Infinium* microarrays^30^.

**Fig. 1:**
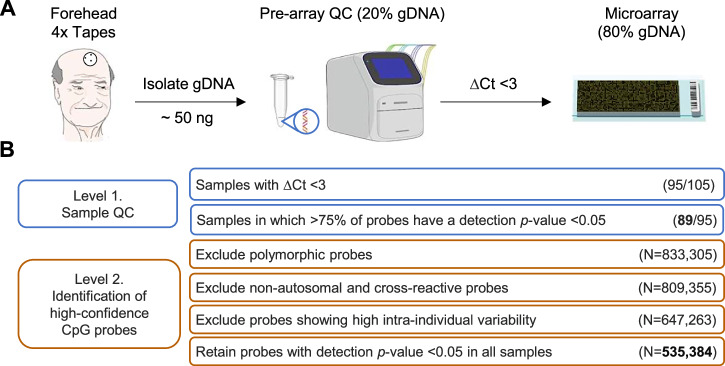
Overview of the TapeLift protocol. **A** Workflow of epidermal sample collection, gDNA isolation and qPCR-based QC to assess the integrity of the material obtained with the adhesive tapes. For quantification and integrity evaluation, around 20% of the gDNA extracted from donors may be utilized. Only samples with a ∆Ct <3 were considered suitable for subsequent *Infinium* microarray analysis. Icons used in this panel were adapted from bioicons.com (CC BY 3.0 & 4.0, CC0): Servier, DBCLS, Marnie-Maddock, KeHan, Marcel Tisch. **B** Computational workflow used for the identification of the high-performance TapeLift CpG probe set. Out of the *N* = 95 samples with ∆Ct <3, *N* = 89 (94%) passed the QC step post-array and were further analyzed. Subsequent probe subsetting finally retained a total of 535,384 high-confidence CpG probes across the genome.

As array-based DNA methylation analyses are strongly dependent on gDNA quality, we incorporated stringent quality control (QC) steps at different stages of the workflow, both before and after EPIC microarray processing (Fig. 1). Critically, we used a qPCR-based assay that was originally developed to assess the level of gDNA integrity in samples from formalin-fixed paraffin-embedded (FFPE) tissue (see Methods). This method compares the threshold cycle (Ct) value of the sample DNA with that of an intact reference DNA, using a difference (ΔCt) > 5 cycles as a cutoff point for the DNA that is considered unsuitable for further processing of FFPE samples. In our cohort of samples, the mean ΔCt was 1.3 cycles, with a maximum of 4.0 cycles (Table S1). As such, our protocol was in principle able to procure sufficient quantities of high-quality DNA for methylation analysis on EPIC arrays. However, due to the limited quantity of DNA recovered from some donors, we lowered the cutoff for further processing of TapeLift samples to ΔCt <3 (Fig. 1A and S1A).

Subsequent analysis of the methylation data obtained from the remaining 95 samples revealed that detection *p*-values were relatively high for a substantial fraction of probes across various datasets. We therefore implemented an additional post-array QC step, by excluding datasets in which >25% of probes had a detection *p*-value > 0.05. This step excluded 6% of the DNA methylation datasets and retained *N* = 89 for further analysis (Fig. S1B and Table S1).

### Identification of a high-confidence TapeLift CpG probe set for downstream analyses

Methylation arrays are based on arrayed primer extension^39^, with primer performance being inherently heterogeneous. While this variation is negligible when large quantities of high-quality input gDNA can be used, low-input and degraded gDNA would result in increasing numbers of failed primer extensions, particularly impacting probes designed for complex genomic areas, such as repetitive sequences or regions with high G + C content. To identify the most robust, high-performance CpG probe set for the gDNA samples obtained with the TapeLift protocol, we followed a systematic serial subsetting approach (Fig. 1B). Thus, after excluding all confounding single-nucleotide polymorphism (SNP)-related probes^30^, non-autosomal probes^40^ and cross-reactive CpG probes^30^, we generated a dataset based on repetitive sampling from the same donors (*N* = 12 samples from *N* = 4 different donors) (Table S2). This allowed us to filter out all CpG probes with limited performance based on their high intra-individual variability (β-value variance >0.1 in the datasets of at least two of the four donors). In a final step, we retained only probes with a detection *p*-value < 0.05 in all datasets, thus establishing a high-performance TapeLift subset of *N* = 535,384 probes (Fig. 1B and Table S3).

**Fig. 2:**
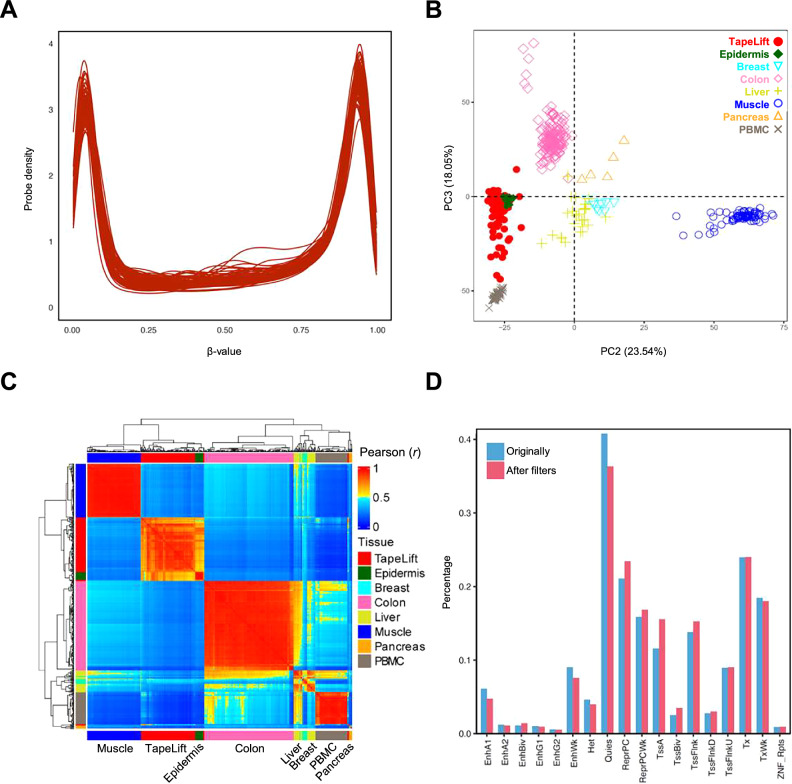
Validation of the TapeLift-derived methylomes. **A** Distribution of methylation beta values (β-values) in the TapeLift datasets. The methylation level of each high-performance *Infinium* probe can range from zero (β-value = 0; completely unmethylated CpG) to one (β-value = 1; fully methylated). The bimodal distribution observed in the plot reflects the high quality of the generated methylomes. **B** PCA performed on the methylation data of the CpGs selected for the methodology clusters the TapeLift-derived datasets together with the methylomes of epidermal samples obtained using suction blisters (from young and old skin). In contrast, the methylomes of other tissues are distinctly separated. **C** Hierarchical clustering of all methylomes included in the PCA shown in (B), based on Pearson correlation values computed from the CpG probes that best distinguish organs and tissues according to the DNA methylation atlas of Loyfer et al. (2023)^34^. This clustering groups all epidermal‑derived samples together while clearly separating them from the other tissues. **D** Genome-wide distribution of the high-confidence CpG probes defined using the TapeLift methodology (red bars) with respect to all probes in the *Infinium* MethylationEPIC v2 array (blue bars). Each CpG probe was assigned to its corresponding genomic compartment using the keratinocyte-specific chromatin states available in the EpiMap project^62^. EnhA active enhancers, EnhBiv bivalent enhancers, EnhG genic enhancers, EnhWk weak enhancers, Het heterochromatin, Quies quiescent/low, ReprPC repressed Polycomb, ReprPCWk repressed Polycomb weak, TssA active transcription start sites, TssBiv bivalent transcription start sites, TssFlnk flanking transcription start sites, TssFlnkU flanking transcription start sites upstream, TssFlnkD flanking transcription start sites downstream, Tx strong transcription, TxWk weak transcription, ZNF/Rpts Zinc-finger genes and repeats.

To assess the quality of the resulting methylomes, we analyzed the distribution of methylation levels for the high-performance probes. This revealed the clear bimodal distribution that is typical for high-quality datasets (Fig. 2A). Moreover, principal component analysis (PCA) of the methylation patterns obtained with the high-performance probe set showed that the TapeLift samples clustered closely with epidermal suction blister samples (Fig. 2B). In contrast, methylation patterns from other healthy human tissues, such as muscle, pancreas, colon, breast, liver, and blood, were distinctly separated in this analysis (Fig. 2B). Additional comparison of the methylomes of TapeLift samples with those from epidermal suction blisters and the same set of tissues, using a recent DNA methylation atlas that includes FACS-purified keratinocyte-specific profiles^34^, further corroborated their tissue-specific identity (Fig. 2C and S2). Importantly, the high-performance probe set largely retained the design structure of the *Infinium* MethylationEPIC v2 array (Fig. 2D), thus allowing for a wide range of potential downstream applications. Taken together, these findings establish the TapeLift protocol as a robust method for the non-invasive analysis of epidermal methylomes.

### Development of DNA methylation clocks to determine epigenetic donor age

**Fig. 3:**
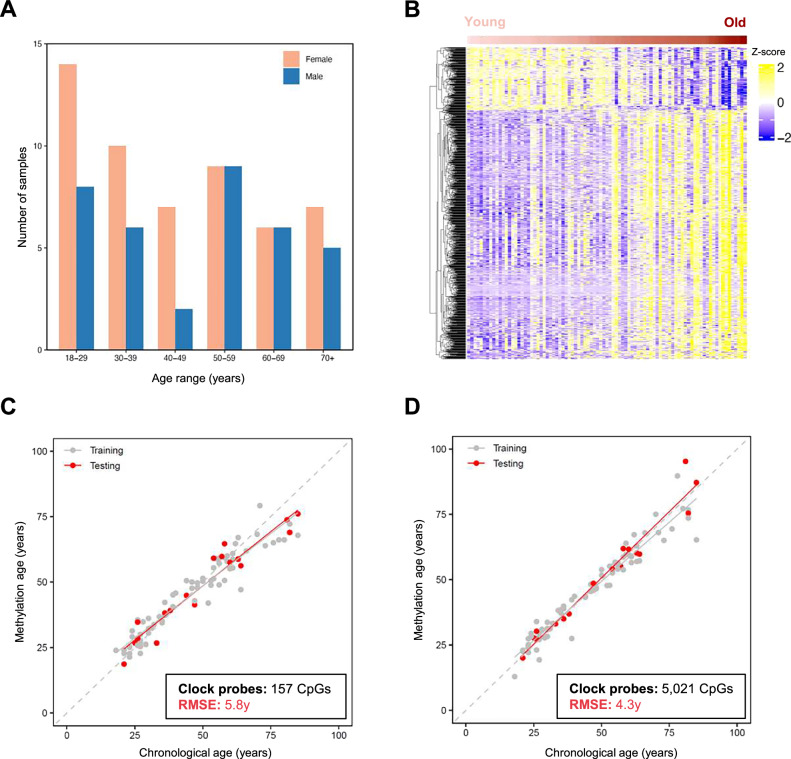
Epigenetic age prediction using TapeLift. **A** Gender and age distribution of donors whose samples successfully passed both QC steps of the protocol. **B** Heatmap illustrating the methylation status of the top 1000 age-associated CpG probes with the highest β-value variance across TapeLift-derived methylomes, stratified by donor chronological age (Benjamini-Hochberg FDR < 0.01). Each row corresponds to a probe, while each column represents a methylome, ordered from youngest to oldest donor. The color code from blue to yellow represents standardized methylation levels (Z-scores). **C** Correlation between the chronological age of each donor and the age predicted by a DNA methylation clock specifically trained on the TapeLift-derived datasets. Samples from the training set (80%, *N* = 71) and the testing (validation) set (20%, *N* = 18) are indicated in different colors. The number of CpGs used to train the clock, along with the RMSE and linear fits (red and gray lines) are shown **D** Correlation between the chronological age of each donor and the age predicted by the DNA methylation clock specifically trained on the TapeLift datasets following a PC-based approach. Samples from the training set (80%, *N* = 71) and the testing (validation) set (20%, *N* = 18) are indicated in different colors. The number of CpGs used to train the clock, along with the RMSE and linear fits (red and gray lines) are shown.

To evaluate the potential of TapeLift-derived methylomes for molecular phenotyping, we used epigenetic age assessment based on DNA methylation clocks. Our initial dataset comprised 53 samples from female donors and 36 samples from male donors, with donor ages ranging from 18 to 85 years (Fig. 3A and Table S1). Analysis of their age-dependent methylation changes detected a subset of 1,000 CpG probes that effectively stratify samples according to the donors’ chronological age (Benjamini-Hochberg FDR < 0.01) (Fig. 3B), suggesting the ability of TapeLift datasets to support the training of DNA methylation clocks. However, established DNA methylation clocks, such as Horvath’s Pan-tissue^41^ and Skin&Blood^20^ clocks, were highly inaccurate with TapeLift datasets (Fig. S3). This was likely attributable to the loss of critical information from filtered‑out low‑performing probes, which affected 73 of 353 CpGs (21%) in Horvath’s Pan‑tissue clock and 138 of 391 CpGs (35%) in the Skin&Blood clock.

To develop a methylation clock specifically adapted to the TapeLift protocol, the methylation dataset was randomly partitioned into a training set, comprising 80% of the samples (*N* = 71), and a validation set, consisting of 20% of the samples (*N* = 18). Clock training using Elastic Net regression resulted in the identification of 157 CpGs that predicted donor age with substantial accuracy (root mean squared error (RMSE) = 6.0 years; Fig. 3C and Table S4). When applied to the validation dataset, this clock demonstrated a comparable performance (RMSE = 5.8 years; Fig. 3C).

Given the susceptibility of first-generation epigenetic clocks to inter-sample noise and variability, we subsequently developed a principal component (PC)-based clock^42^ to more reliably capture consistent, age-associated methylation patterns. This methodological refinement led to the identification of 5021 CpGs that predicted donor age with increased accuracy (RMSE = 4.7 years; Fig. 3D and Table S5). Application of this clock to the validation dataset yielded a prediction accuracy of 4.3 years (Fig. 3D).

To further validate and assess the robustness of the two clocks derived from TapeLift datasets, we generated an independent dataset of 19 new donors (ten females and nine males spanning diverse ages; see Table S6). In this cohort, both clocks again predicted donor age with comparable accuracy (RMSE = 5.1 and 6.2 years, respectively; Fig. S4). These findings underscore the potential of TapeLift-derived methylomes for highly precise epigenetic age prediction, thereby highlighting their broader utility in molecular phenotyping.

### Evaluation of skin cancer markers

**Fig. 4:**
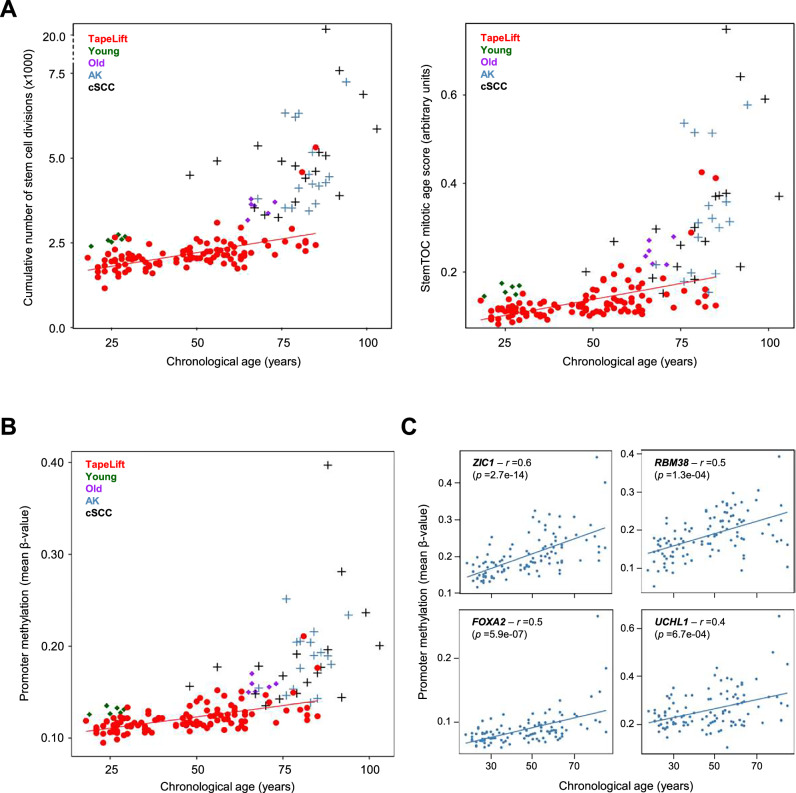
TapeLift-based evaluation of cancer biomarkers. **A** Mitotic rate of the TapeLift-derived epidermal samples, suction blister-derived healthy epidermis samples (from young and old skin), precancerous AK samples and cSCC tumor samples, ordered by chronological age. Mitotic rates were calculated using the EpiTOC2 clock^23^ (left panel) and the StemTOC clock^24^ (right panel), respectively. The line depicts the degree of correlation between chronological age and mitotic age for the TapeLift methylomes. **B** Mean promoter methylation level in TapeLift- and suction blister-derived (young and old) healthy epidermal datasets, as well as in AK and cSCC methylomes, for the 50 tumor suppressor genes (TSGs) that showed the most pronounced hypermethylation (*p*-value < 0.05) between young (18–29 years) and older ( ≥ 60 years) donors. All samples are ordered by chronological age. The line illustrates the correlation between chronological age and the age-associated gain in promoter methylation at the TSGs. **C** Promoter methylation levels of particular examples of TSGs from (B), and with a published link to epithelial tumorigenesis. Samples are ordered by chronological age. For each TSG, the Pearson correlation coefficient (*r*) between age and promoter methylation levels is calculated, with a linear correlation line included for visual reference.

Finally, we assessed the utility of the TapeLift protocol in monitoring DNA methylation changes associated with skin cancer. First, we calculated the mitotic age of all 108 TapeLift-derived samples by estimating the cumulative number of stem cell divisions for the corresponding epidermal cells. This was performed using the epiTOC2 and stemTOC algorithms, which have been shown to detect increasing mitotic rates in preneoplastic lesions and tumors relative to healthy tissue^23,24^. We also integrated the available healthy epidermal samples obtained through suction blistering from a cohort of six younger and six older donors (Fig. 2B, C), along with multiple biopsy-derived epidermis samples from cSCC and its primary precursor lesion, AK^17,43^, as references. Our findings indicate a progressive increase in mitotic age correlating with chronological aging in both TapeLift-derived samples and suction blister-derived methylomes (Fig. 4A). Notably, this division rate is even more elevated in AK and cSCC methylomes, highlighting distinct proliferative dynamics associated with malignant progression.

Hypermethylation of tumor suppressor gene (TSG) promoters is a well-known hallmark of KCs and other cancers^8,44,45^. Our initial analysis comparing the methylation status of promoter regions across 859 TSGs^46^ showed varying degrees of similarity between TapeLift samples and AK and cSCC samples, with some of the older samples reaching remarkably high correlation values (Fig. S5A). Further investigation into the 50 TSGs that exhibited the most pronounced promoter hypermethylation between the epidermal methylomes of young and old donors (Fig. S5B and Table S7) revealed a progressive methylation gain in KC samples (Fig. 4B). Notably, the cancer-promoting effects of epigenetic silencing for several of these TSGs, including *ZIC1*, *RBM38*, *UCHL1* and *FOXA2*, are well-documented in epithelial tumors^47–52^, underscoring their potential relevance as KC biomarkers (Fig. 4C). Additionally, *ID4*, another TSG exhibiting an age-associated increase in promoter methylation in the TapeLift datasets (Table S7), is known to undergo UVR-induced silencing that directly contributes to cSCC development^53^. Taken together, our analysis of several DNA methylation-based KC markers indicates that TapeLift samples contain information that could be used to evaluate the risk of epidermal tumor formation.

## Discussion

Tape stripping has been successfully used for the recovery of epidermal RNA, proteins, lipids, and microbial communities^25–27^. However, the low yield and instability of these biomolecules has limited the application potential of the sampling method. Similarly, the application of tape stripping for the analysis of gDNA has presented significant challenges, primarily due to the low yield and quality of the DNA retrieved from keratinocytes in the superficial epidermal layers. These limitations also extended to DNA methylation profiling, thus curtailing its considerable potential for assessing skin health and skin cancer risk. Furthermore, the widely used *Infinium* microarray requires a few hundred nanograms of high-quality DNA for optimum performance, which is usually not obtainable by tape stripping. As a result, the only genome-wide DNA methylation study performed to date using tape stripping required PCR-based amplification of the obtained DNA, a step prone to introducing artifacts^29^, and next-generation sequencing, which is considerably more expensive and complex to analyze^28^.

Our TapeLift protocol successfully overcomes these challenges and, for the first time, enables the reliable generation of high-quality epidermal methylomes using DNA methylation microarrays. This breakthrough is based on the integration of several key components: (1) highly efficient adhesive strips for the detachment of keratinocytes, (2) an efficient DNA isolation protocol optimized for low-input material conditions, and (3) a computational workflow based on the *Infinium* probes that demonstrate acceptable performance under low-input conditions. The entire process was also reinforced by key QC steps to ensure methodological robustness and reliability.

As for the collection of biological material, it is essential to underscore the performance of the adhesive tapes used in this methodology. While previous approaches required eight tapes from a different manufacturer^28^, our protocol was performed with only four tapes. Interestingly, parallel tests also yielded substantial quantities of high-quality gDNA with two tapes (*N* = 10) and one tape (*N* = 10), respectively (Table S8). Analysis of methylomes obtained from 1-2 tapes (*N* = 5) demonstrated comparable quality (Fig. S6; Table S9), indicating that the sampling protocol can be further optimized with fewer tapes. Thus, although other tapes may also be effective for obtaining gDNA via tape stripping, our tape appears particularly suitable for the removal of epidermal keratinocytes from the outermost skin layers. Aiming to reduce costs and simplify the procedure, our methodology also employs a conventional gDNA isolation protocol without columns or magnetic beads. However, several adaptations were implemented to maximize yield and minimize DNA loss during the process, including overnight cell lysis and the use of a co-precipitant (see Methods).

Our study illustrates how the integrity of the extracted gDNA is a critical parameter to determine the successful performance of low-input DNA methylation microarrays. Even though some of our samples contained less than ten nanograms of DNA, the methylation distribution of probes exhibited the classical bimodal distribution, indicating good array performance. While previous studies have managed to generate microarray-derived datasets using only 1 ng of material, the DNA came from serial dilutions of highly pure extractions from sorted cells^30^. In contrast, our gDNA preparations likely contain degraded DNA and DNA from the skin microbiome, which may further reduce the proportion of intact human gDNA. Our results show that a focused analysis based on high-performing CpG probes can overcome many of these obstacles, with TapeLift-derived methylomes being similar to methylomes from reference epidermis samples and/or sorted keratinocytes.

After verifying that healthy TapeLift-derived methylomes retained age-dependent information, we initially attempted to apply two well-established first-generation epigenetic clocks: the original Pan-tissue^41^ and Skin&Blood^20^ clocks. Neither clock was able to accurately predict donor age, likely due to the poor performance of key clock probes under low-input conditions. However, after training novel clocks on TapeLift datasets, we were able to accurately predict donor ages with a prediction error of only 4.3 years, which is comparable to the best-performing epigenetic clocks^18^. Importantly, the application of these clocks identified certain outliers with notably increased or decreased epigenetic ages. Future research will be needed to address whether these outliers present a distinct skin aging phenotype based on dermatological parameters.

DNA methylation microarrays play a major role in cancer research, including studies on KCs^8,43^. The MethylationEPIC v2 array used in this study also includes 824 non-variable (nv) probes capable of detecting different mutations in cancer-associated genes^30^. Notably, among the probes retained in the high-confidence subset of the TapeLift datasets (Table S3), several exhibited significant alterations in specific samples, potentially reflecting the presence of genetic mutations that could contribute to tumor formation (Fig. S7). This may indicate further opportunities to develop integrated biomarkers for skin cancer risk prediction. However, optimal implementation would require the design of a microarray incorporating a larger number of nv probes specifically targeting skin cancer. Similarly, our findings on TSGs undergoing epigenetic silencing during epidermal aging, along with data obtained from epiTOC2^23^ and stemTOC^24^ further underscore the potential of the TapeLift protocol for the development of epigenetic skin cancer risk predictors. Silencing of TSGs, such as *ZIC1, RBM38, UCHL1 and FOXA2* is known to play an important role in epithelial tumorigenesis^47–52^, and UVR-induced promoter hypermethylation and silencing of *ID4* directly contribute to cSCC development^53^. Furthermore, epiTOC2 and stemTOC consistently indicate elevated cell division rates in paratumoral or precancerous tissues relative to healthy samples, with an even higher mitotic rate observed in tumors compared to adjacent paratumoral regions^23,24^. This pattern aligns with the increased mitotic activity predicted for aged epidermal methylomes, regardless of whether they were obtained via TapeLift or suction-blister sampling.

While the translation of these epigenetic parameters into a concrete KC risk stratification approach remains to be accomplished, an integrated analysis of all parameters studied in our full set of TapeLift‑derived methylomes suggests that epidermal samples with accelerated epigenetic age may tend to exhibit a higher mitotic ratio and increased TSG promoter methylation (Fig. S8). The results of our study therefore lay the foundation for the broad adoption and application of DNA methylation profiling in non‑invasive skin health monitoring.

## Methods

### Samples

TapeLift samples were collected from the forehead of 105 Caucasian donors of both genders, aged 18–85 years, and with Fitzpatrick skin types I–IV. All voluntary donors self-reported no known history of dermatological disease. The study was approved by the Ethics Committee of the Faculty of Medicine at the University of Heidelberg (ref. S-783/2024), and all participants provided written, informed consent. A comprehensive overview of all TapeLift samples is presented in Tables S1, S2, S6 and S8.

Healthy epidermal methylomes obtained via suction blisters (*N* = 6 young and *N* = 6 old samples) are publicly available at the ArrayExpress repository (E-MTAB-5738)^17^. Methylomes of colon, muscle, pancreas, and all other tissues and cell types used to confirm the cutaneous origin of the TapeLift datasets, can be accessed through the Gene Expression Omnibus (GEO) repository under the following accessions: GSE186458^34^, GSE199057^54^, GSE134217^55^, GSE244359^56^, GSE268211^57^, GSE213029^58^, GSE100850^59^, GSE198627^60^, and GSE132181^61^. Additionally, keratinocyte cancer datasets used to investigate cancer-related DNA methylation changes in TapeLift methylomes are available in the ArrayExpress repository under the accessions E-MTAB-5738^17^ and E-MTAB-11856^43^.

### Epidermal tape stripping

Initially, the forehead region of interest was cleaned with 70% ethanol, and a 2.0 × 2.5 cm² square was delineated using a standard pen. A square piece of polyurethane film with an advanced polyacrylate adhesive coating (Beiersdorf AG, custom product), was then applied to the marked area. After applying consistent pressure for 5–10 s, the strip was carefully removed with clean forceps and placed into a sterile 50 mL Falcon tube. This process was repeated three additional times on the same skin region, with all four strips collected into the same tube for subsequent gDNA isolation, either immediately or within 15 days.

### gDNA isolation

For gDNA extraction, 2.5 mL of lysis buffer (10 mM TRIS-HCl, pH 8.0; 5 mM EDTA; 100 mM NaCl; 1% SDS (w/v); 100 µg/mL Proteinase K; 40 µg/mL RNAse A) were added to the 50-mL Falcon tube containing the four adhesive strips, ensuring complete submersion. The tube was then sealed with Parafilm and incubated overnight at 37 °C. The following morning, the lysate was transferred to a 15-mL Falcon tube, excluding the strips, and supplemented with 1.25 mL of 5 M sodium chloride solution. After vigorous mixing, the solution was aliquoted into three 1.5 mL Eppendorf tubes and centrifuged at room temperature and maximum speed for 8 minutes. The clear supernatants were pooled into a fresh 15-mL Falcon tube, while the precipitates were discarded. In cases where residual debris remained post-centrifugation, an additional centrifugation step was performed until a debris-free supernatant was obtained.

Subsequently, 4 µL of GlycoBlue Co-precipitant (Thermo) were added to the pooled supernatant, followed by brief vortexing. The solution was further mixed with 2.8 mL of isopropanol by inversion (10–15 repetitions) and incubated at -80 °C for 10 min. The entire volume was then divided into four 1.5 mL Eppendorf tubes and centrifuged at 4 °C and maximum speed for 10 minutes, resulting in the formation of small, visible gDNA pellets. The supernatants were carefully removed, and 50 µL of 70% ethanol were added to each tube to wash the precipitated material. The four pellets were detached and pooled into a single Eppendorf tube using the ethanol, followed by centrifugation at 4 °C and maximum speed for 5 min. After discarding the supernatant, the pellet was air-dried for a few minutes to ensure the complete evaporation of residual alcohol. Finally, the dried gDNA pellet was resuspended in 40 µL of ddH₂O.

To quantify the amount of gDNA extracted from each sample, the Quant-iT PicoGreen dsDNA Assay Kit (Thermo) was used, while to assess gDNA integrity, quantitative PCR (qPCR) was performed using the reference sample, primers and reagents included in the *Infinium* FFPE QC and DNA Restoration Kit (Illumina). Samples exhibiting a Ct difference of less than three cycles compared to the high-quality reference DNA (ΔCt <3) were deemed suitable and subsequently used for microarray analysis, without any restoration and following the standard—sodium bisulfite-based—protocol.

### DNA methylation analysis

Genomic DNA was analyzed on Illumina Human MethylationEPIC v2 methylation arrays^30–32^ (DKFZ Microarray Core Facility). Raw IDAT files from each sample were processed using the R (v4.4.1) package SeSAMe (v1.22.2)^40^. Initial QC assessments included evaluation of background signal intensity, mean signal intensity, number of missing values (NAs), and bisulfite conversion efficiency. DNA methylation levels were quantified by calculating β-values from dye bias-corrected and background-substracted probe signal intensities. The β-value represents the proportion of methylation at each CpG site, ranging from 0 (fully unmethylated) to 1 (fully methylated). To assess probe reliability, detection *p*-values were computed using the pOOBAH algorithm, which estimates background signal using out-of-band (OOB) fluorescence intensities. Considering the low input of our DNA samples, only those samples with at least 75% of CpG probes showing a detection *p*-value < 0.05 were retained for further analysis.

Next, we applied multiple filtering steps to select high-confidence CpG probes for further analysis of TapeLift samples. First, probes corresponding to CpGs overlapping with known single nucleotide polymorphisms (SNPs)^30^ were excluded to avoid potential allelic bias. Second, probes located on sex chromosomes^40^ and cross-reactive probes^30^ were subsequently removed. After generating two additional datasets from *N* = 4 donors—one consisting of samples collected from the same location on the donors’ foreheads but on different days, and the other consisting of samples from different yet very close locations on the donors’ foreheads on the same day (Table S2)—CpG probes showing high intra-individual variability (β-value variance >0.1 in the three datasets of at least two of the four donors), attributable only to technical rather than biological factors, were also filtered out. To maximize the exclusion of unreliable probes, we slightly relaxed our pre- and post-array QC criteria when processing these additional datasets. Specifically, we accepted samples with gDNA ΔCt values ≤ 3.5 and retained datasets in which at least 70% of CpG probes exhibited detection *p*-values < 0.05. The final set of probes for the TapeLift protocol (*N* = 535,384) (Table S3) was determined by selecting those with a detection *p*-value < 0.05 across all datasets.

For the comparison of TapeLift samples with those in the recently published DNA methylation atlas^34^, the top 25 cell type-specific unmethylated CpG probes were utilized. Positions of various genomic features (or compartments) in keratinocytes were identified using chromatin state data for this cell type, acquired from the EpiMap project (accessions: BSS01068, and BSS01071)^62^.

To investigate age-related methylation changes in the TapeLift datasets, the limma package was employed to perform linear modeling on β-values^63^, with age included as a continuous predictor. Empirical Bayes moderation was applied to improve variance estimation across CpG sites. CpG sites with Benjamini–Hochberg adjusted *p*-values < 0.01 were considered significantly associated with age. To illustrate methylation dynamics over time, the top 1000 most variable age-associated probes were selected based on β-value variance.

### Training and optimization of the epigenetic age clocks

To develop the first-generation epigenetic clock, designed to predict chronological age based on normalized DNA methylation β-values, we employed an Elastic Net regression model, implemented in the R package glmnet (v4.1.8), with an α parameter of 0.1. This method combines Lasso and Ridge penalties, promoting model sparsity while retaining informative, correlated CpG sites. Model training was performed using ten-fold cross-validation to optimize performance and minimize overfitting. The predictive accuracy of the epigenetic clock was evaluated using the Pearson correlation coefficient and root mean squared error (RMSE) to compare DNA methylation-based predicted ages with observed chronological ages.

In the PC-based clock framework, normalized β-values were replaced by the loadings of principal components, thereby shifting the unit of analysis from individual CpG sites to broader methylation patterns. PCA was conducted using the prcomp function, and selected PCs were used as predictors in the age regression model, following the instructions provided at https://github.com/MorganLevineLab/PC-Clocks/. In this case, we employed an α parameter of 0.5.

### Analysis of epigenetic and non-epigenetic skin cancer markers

To estimate mitotic age and stem cell division rates (SCDR), we applied the epiTOC2 and stemTOC algorithms^23,24^. For epiTOC2, we used the publicly available R script and default parameters, and following the instructions provided at 10.5281/zenodo.2632938. EpiTOC2 scores were computed from the β-values for the relevant CpG sites, as described in the original methodology. For StemTOC, we applied the corresponding function included in the R package EpiMitClocks^24^. In both cases, only the high-confidence probe subset was used, comprising 135 of the 163 EpiTOC2 clock probes, and 280 of the 371 StemTOC clock probes.

For the TSG study, a comprehensive gene list (*N* = 1217) was initially obtained from the TSGene 2.0 database^46^. Of these, only the 859 TSGs whose promoters—TSS1500 and TSS200 regions, according to Illumina annotation—were represented by a minimum of two CpG sites within the high-confidence probe set were considered further. Pearson correlation coefficients were then computed to assess the relationship between samples based on the methylation levels of their promoter CpGs. To examine age-related alterations in the methylation patterns of the TSGs, mean promoter methylation levels of each gene were finally compared between a younger sample subset (18–29 years) and an older sample subset ( ≥ 60 years). TSGs exhibiting significant hypermethylation in the older group (*p*-value < 0.05) were identified, and the 50 genes demonstrating the greatest methylation change were selected for subsequent analysis (Table S7).

Finally, to investigate mutation profiles within the TapeLift datasets, we employed 333 non-variable (nv) probes—targeting allelic variants across 39 cancer-related genes—included in the high-confidence probe subset (Table S3).

## Supplementary information

Supplementary information

Supplementary Tables

## Data Availability

DNA methylation datasets generated for this study have been deposited in the Gene Expression Omnibus (GEO) repository under the accession number GSE304036.
