# Supplementary material for: Non-invasive epidermis sampling for DNA methylation-based prediction of skin cancer phenotypes
Source: NPJ Precis Oncol. 2026 Jan 27;10:89. doi: 10.1038/s41698-026-01302-7 (PMC12948955; doi:10.1038/s41698-026-01302-7)
Supplement: Supplementary file 1 — Supplementary information [file 41698_2026_1302_MOESM1_ESM.docx]

**Supplementary information**

**Non-invasive epidermis sampling for DNA methylation-based prediction of skin cancer phenotypes**

Rodríguez-Paredes *et al*.

**Contents:**

Supplementary figures S1-S8

Supplementary tables S1-S9 (Excel file)

Supplementary references

**Supplementary figures**

**
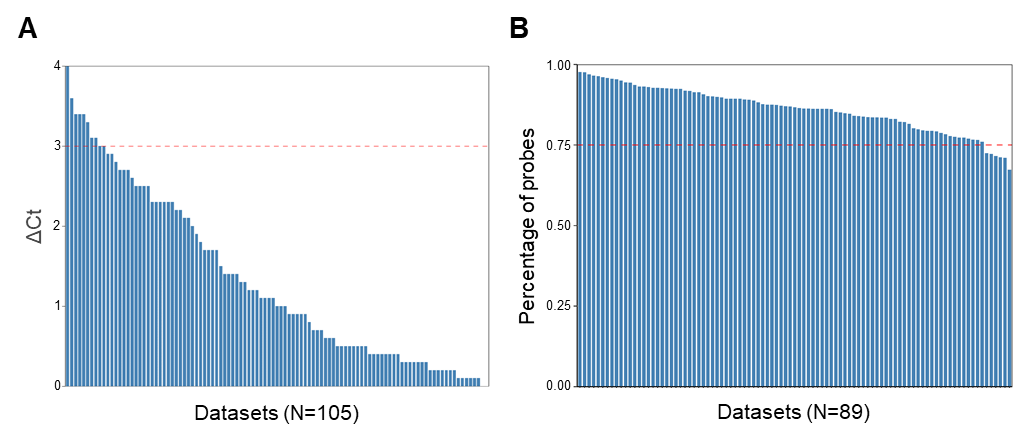
**

**Figure S1. Quality control (QC) parameters for the TapeLift datasets. (A)** Proportion of datasets exhibiting ΔCt <3. **(B)** Proportion of probes exhibiting detection *p*-values <0.05 within the datasets meeting the ΔCt <3 criterion. In both panels, the red dashed lines indicate the thresholds used in the pre- and post-array QC steps.


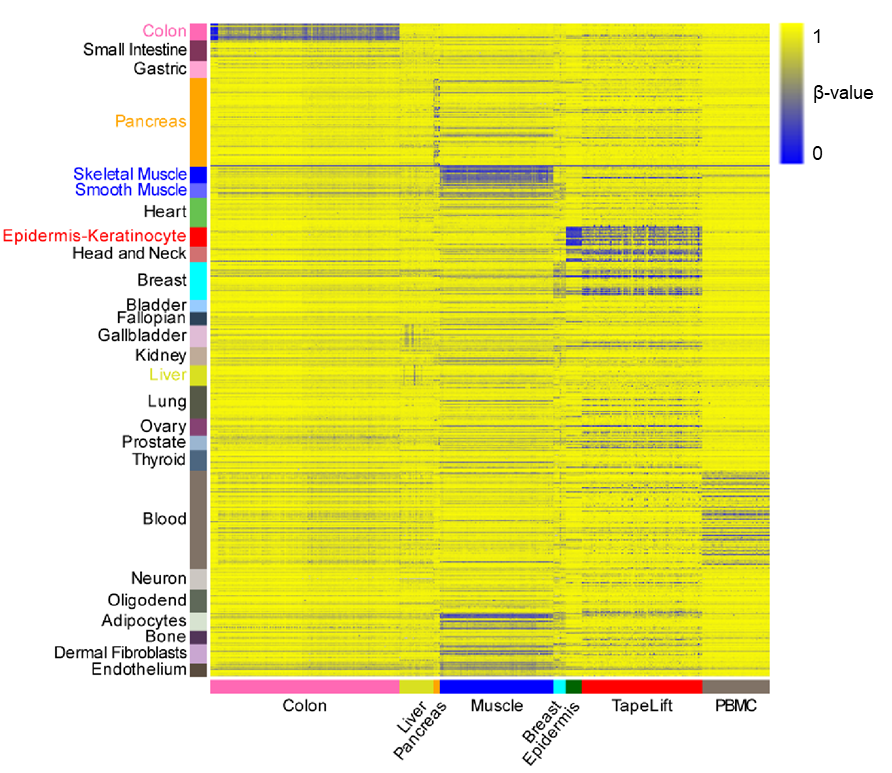


**Figure S2. Classification of TapeLift-derived epidermal methylomes, epidermal suction blister-derived methylomes and methylomes from six unrelated tissues.** The heatmap is based on the top 25 cell type/tissue-specific unmethylated CpG probes from each of the organs and tissues indicated on the left^1^. The color code from blue to yellow represents the methylation level of each probe in β-values, ranging from 0 (completely unmethylated CpG) to 1 (fully methylated). PBMC: peripheral blood mononuclear cells.

**
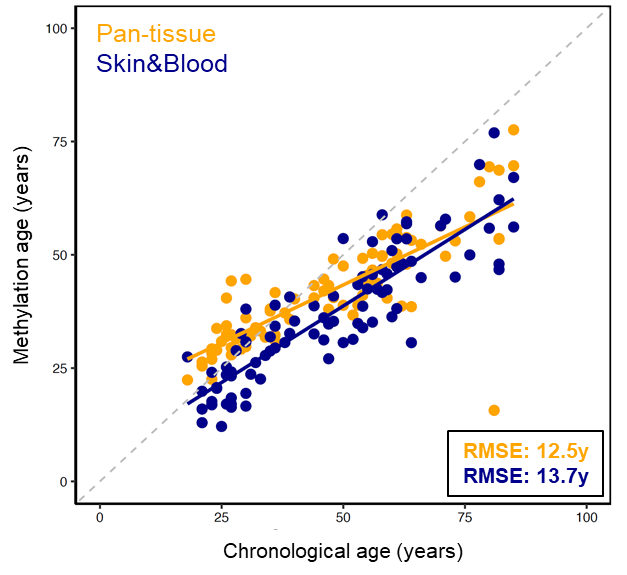
**

**Figure S3. Age estimation of donors from TapeLift-derived samples (N=89) using Horvath’s Pan-Tissue^2^ and Skin & Blood^3^ epigenetic clocks.** The plot displays the linear fit achieved for each clock relative to the ideal correlation (grey dashed line). Mean prediction error for each clock is also presented.


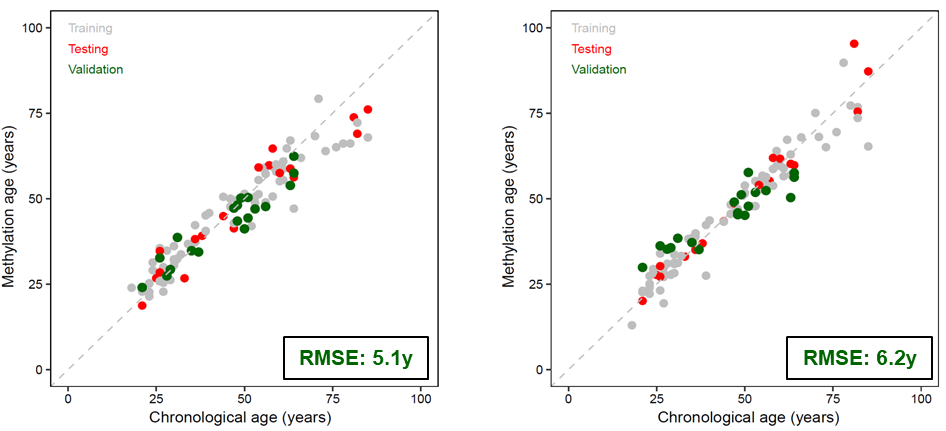


**Figure S4. Validation of the TapeLift‑derived epigenetic clocks in independent samples.** Age predictions for an independent cohort of 19 donors using the traditionally constructed (left) and PC‑based (right) clocks. Grey dashed lines in the plots indicate the ideal correlation. The mean prediction error for each clock is shown within each graph.

**
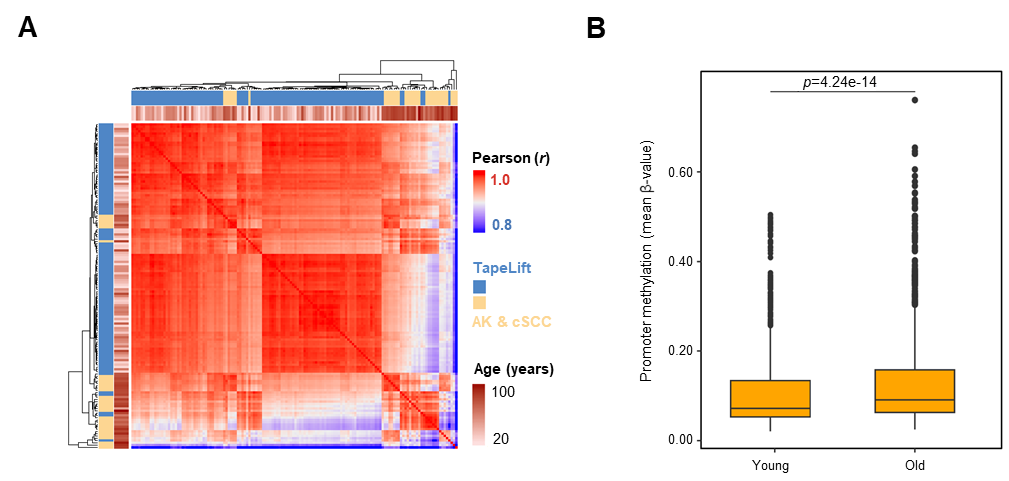
**

**Figure S5. Promoter methylation status of TSGs in TapeLift datasets. (A)** Hierarchical clustering illustrating Pearson correlation (*r*) among TapeLift-derived healthy epidermis and KC samples (AK and cSCC), based on DNA methylation levels of the CpGs in the 859 tumor suppressor genes (TSGs) analyzed in the study. **(B)** Boxplots comparing the promoter methylation levels of the 50 TSGs showing the most significant differential methylation (*p*-value <0.05) between the younger (18–29 years) and older (≥60 years) epidermal samples.

**
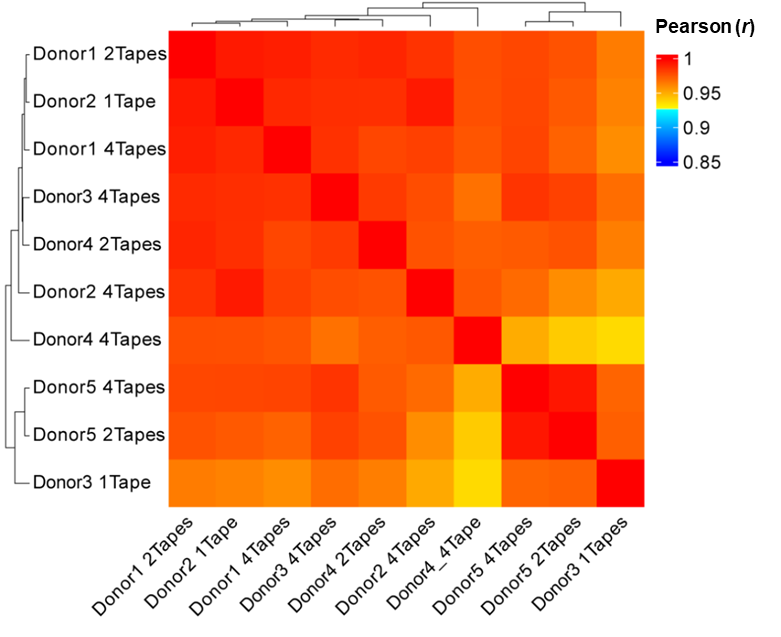
**

**Figure S6. Comparison of Tapelift-derived methylomes obtained with one or two tapes versus four.** The heatmap shows strong similarity among all methylomes, based on probe-by-probe Pearson correlations across all CpGs, indicating comparable quality regardless of the number of tapes used for sampling. Donor 1: samples 127 (two tapes) and 18 (four tapes); donor 2: samples 125 (one) and 96 (four); donor 3: samples 126 (one) and 6 (four); donor 4: samples 129 (two) and 5 (four); donor 5: samples 128 (two) and 8 (four).

**
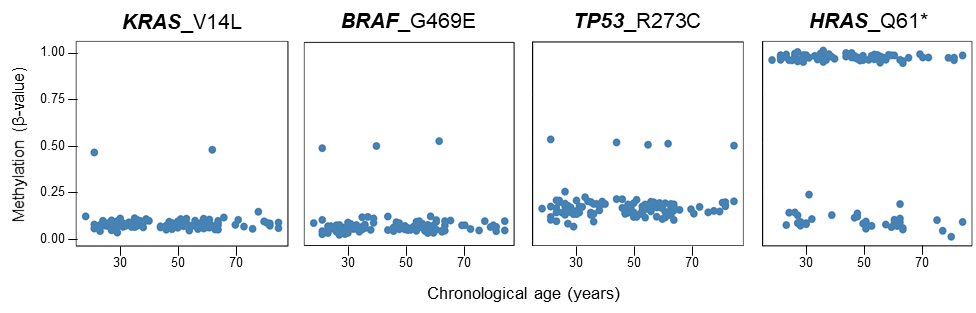
**

**Figure S7. Assessment of somatic mutations in TapeLift datasets based on changes in methylation status of non-variable (nv) probes included in the MethylationEPIC v2 array.** As an example, analysis of four specific mutations in key cancer-related genes that contribute to tumor development and/or progression is shown.

**
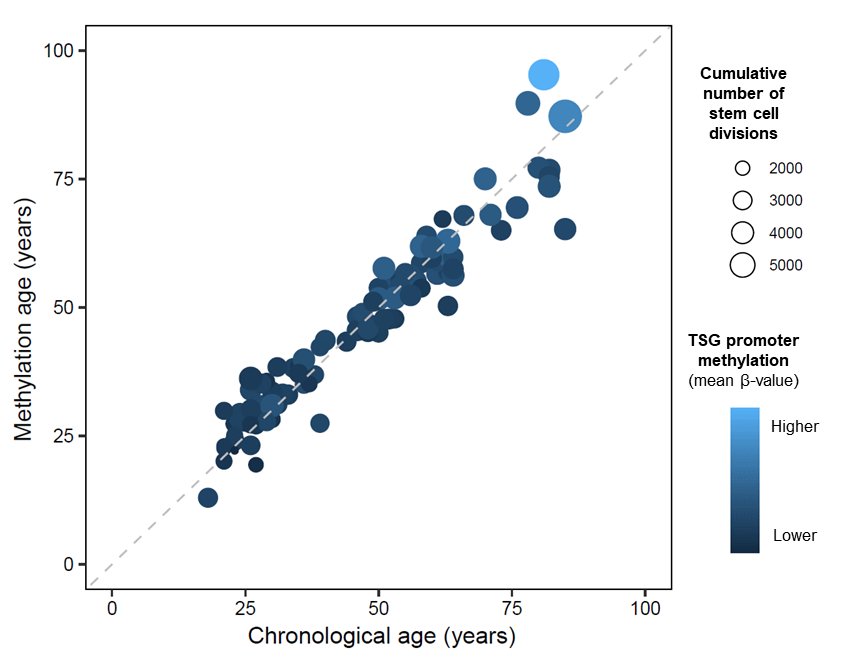
**

**Figure S8. Integrated visualization of chronological age, epigenetic age, mitotic rate, and TSG promoter methylation in TapeLift‑derived epidermal samples.** The epigenetic age shown in the Y axis corresponds to the estimate generated by the PC‑based DNA methylation clock. The grey dashed line indicates the ideal correlation.

**Supplementary tables** (Excel file)

**Table S1. Donor information, QC results of TapeLift samples and predicted DNA methylation ages - Main dataset.**

**Table S2. General overview of the TapeLift samples used to exclude CpG probes exhibiting high intra-individual variability.**

**Table S3. Final set of high-confidence CpG probes for the TapeLift methodology (N=535,384).** Non-variable (nv) probes that can be used for mutation assessment are highlighted in bold.

**Table S4. CpG probes forming the first-generation epigenetic clock specifically developed for the TapeLift methodology (N=157).**

**Table S5. CpG probes forming the PC-based epigenetic clock specifically developed for the TapeLift methodology (N=5,021).**

**Table S6. Donor information, QC results of TapeLift samples and predicted DNA methylation ages - Independent validation dataset.**

**Table S7. Top 50 TSGs whose promoter regions exhibit the most statistically significant differences in methylation between the TapeLift-derived methylomes of the youngest (18–29 y/o) and oldest (>60 y/o) donors.** The last column presents the correlation between donors’ chronological age and the average promoter methylation levels derived from their respective TapeLift datasets.

**Table S8. QC parameters obtained using one or two tapes instead of the four applied for this study.**

**Table S9. Donor information and QC results of TapeLift datasets obtained with one or two tapes.**

**Supplementary references**

1. Loyfer N, Magenheim J, Peretz A, Cann G, Bredno J, Klochendler A, *et al*.: **A DNA methylation atlas of normal human cell types.** *Nature* 2023, **613:**355-364.
2. Horvath, S. **DNA methylation age of human tissues and cell types**. *Genome Biol* **14**, R115 (2013).
3. Horvath, S. et al. **Epigenetic clock for skin and blood cells applied to Hutchinson Gilford Progeria Syndrome and ex vivo studies.** *Aging (Albany NY)* **10**, 1758-1775 (2018).
